# Psychiatrists in psycho-cardio teams in practice

**DOI:** 10.1192/j.eurpsy.2026.10404

**Published:** 2026-07-31

**Authors:** M. Rojnic Kuzman

**Affiliations:** 1 Zagreb School of Medicine; 2 Zagreb University Hospital Centre, Zagreb, Croatia

## Abstract

**Abstract:**

In 2025, the European Society of Cardiology (ESC) issued a pivotal document: the *Clinical Consensus Statement on Mental Health and Cardiovascular Disease*, developed by the ESC Task Force on Mental Health and Cardiovascular Disease under the auspices of the ESC Clinical Practice Guidelines Committee. It was endorsed by the European Federation of Psychologists’ Associations (EFPA), the European Psychiatric Association (EPA), and the International Society of Behavioral Medicine (ISBM).

The document highlights the bidirectional relationship between mental and cardiovascular health, emphasizing its impact on cardiovascular risk and prognosis. It offers evidence-based recommendations for assessing and managing mental health conditions in people at risk for, or living with, cardiovascular disease.

Practical guidance includes integrating mental health into routine cardiology through psycho-cardiac teams, with psychiatrists as integral members. Using a stepwise clinical approach, psychiatrists assess suspected mental health disorders, provide comprehensive pharmacological and psychosocial treatment, and guide care for patients with pre-existing mental disorders who develop cardiovascular disease.

Implementing these recommendations structurally could represent a paradigm shift toward a truly whole-person approach in both cardiovascular and psychiatric care, ensuring that mental and cardiovascular health are addressed in tandem.

**Disclosure of Interest:**

None Declared

